# ASCU-Net: Attention Gate, Spatial and Channel Attention U-Net for Skin Lesion Segmentation

**DOI:** 10.3390/diagnostics11030501

**Published:** 2021-03-12

**Authors:** Xiaozhong Tong, Junyu Wei, Bei Sun, Shaojing Su, Zhen Zuo, Peng Wu

**Affiliations:** College of Intelligence Science and Technology, National University of Defense Technology, Changsha 410073, China; tongxiaozhong@nudt.edu.cn (X.T.); sunbei08@nudt.edu.cn (B.S.); ssjing@nudt.edu.cn (S.S.); z.zuo@nudt.edu.cn (Z.Z.); pengwu@nudt.edu.cn (P.W.)

**Keywords:** skin lesion segmentation, U-Net, attention mechanism, deep convolutional neural networks

## Abstract

Segmentation of skin lesions is a challenging task because of the wide range of skin lesion shapes, sizes, colors, and texture types. In the past few years, deep learning networks such as U-Net have been successfully applied to medical image segmentation and exhibited faster and more accurate performance. In this paper, we propose an extended version of U-Net for the segmentation of skin lesions using the concept of the triple attention mechanism. We first selected regions using attention coefficients computed by the attention gate and contextual information. Second, a dual attention decoding module consisting of spatial attention and channel attention was used to capture the spatial correlation between features and improve segmentation performance. The combination of the three attentional mechanisms helped the network to focus on a more relevant field of view of the target. The proposed model was evaluated using three datasets, ISIC-2016, ISIC-2017, and PH2. The experimental results demonstrated the effectiveness of our method with strong robustness to the presence of irregular borders, lesion and skin smooth transitions, noise, and artifacts.

## 1. Introduction

### 1.1. General Background

Skin cancer is one of the most common and deadly cancers. In 2020, the American Cancer Society reported that there will be approximately 100,350 new cases of melanoma and about 6850 people will die from this cancer [1]. Non-melanoma cancers are also responsible for a large number of deaths. The World Health Organization (WHO) reported that 2–3 million non-melanoma skin cancers and 132,000 melanoma skin cancers occur globally each year [2]. However, with early detection and diagnosis, melanoma can be simply excised to ensure full recovery. Survival rates exceed 95% in cases of early diagnosis and less than 20% in cases of late detection [3]. Therefore, accurate analysis of medical images is important for early diagnosis and treatment of skin diseases. 

**Figure 1:**
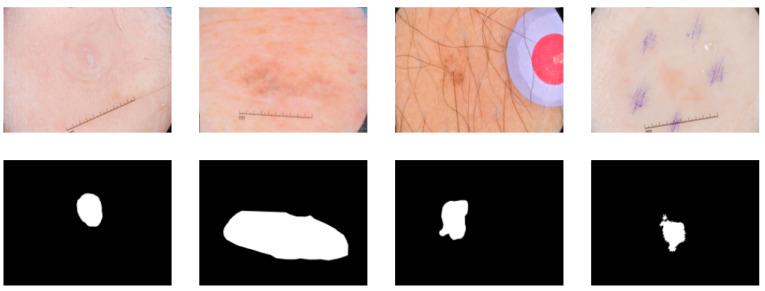
Several examples of skin lesions that are difficult to isolate accurately.

In order to make melanoma detection more accurate and reliable, dermoscopy is widely used for the non-invasive early diagnosis of this disease. However, this strategy for the detection of melanoma may be inaccurate or subjective, based on the experience of dermatologists alone [4]. In recent years, with the development of computer vision, medical image segmentation has become an important part of computer-aided diagnosis, which can support physicians in diagnosing dermoscopic images with speed and accuracy [5,6], providing professional interpretation of medical images [5]. However, segmentation of skin cancers is a challenging task because of the low image contrast and differences in color and size of skin lesions as well as the presence of air bubbles, hair, and ebony frames [7]. Figure 1 shows a partial image of a skin lesion that is difficult to segment accurately when similar to the background. As a result, deep learning algorithms need to achieve a high level of accuracy in order to perform well in skin lesion segmentation tasks.

In earlier times, the segmentation of skin lesions obtained using traditional methods was unsatisfactory. In recent years, different types of deep convolutional neural networks (CNN) have been widely used in various fields [8], especially for medical image segmentation. U-Net [9] is the most common network structure for medical image segmentation, consisting of encoding and decoding paths. Oktay et al. [10] proposed the addition of attention gate (AG) to U-Net skipping connections to improve prediction accuracy and sensitivity in a pancreas segmentation protocol. Guo et al. [11] introduced a spatial attention module in a convolutional neural network for medical image segmentation and detection. Chen et al. [12] focused on explicit relationships between channels and proposed adding spatial channel-wise convolution to the up-sampling and down-sampling modules to improve the image segmentation performance of the network. Li et al. [7] proposed a dense deconvolutional network and U-Net combined for the automatic segmentation of skin lesion segmentation, where multiple dense blocks [13] are stacked together to improve the representativeness of the model. Wang et al. [14] combined the pyramid attention network and U-Net for skin lesion segmentation. Furthermore, attention-based networks have been widely used for different tasks in computer vision [15]. Sun et al. [16] presented the interpretability of SAUNet for spatial and channel attentional mechanisms and demonstrated that attention mechanisms can effectively enhance the robustness of networks. Xu et al. [17] demonstrated that attention can improve the semantic segmentation results of the network.

In conjunction with the latest advances in the attention mechanism, in this paper, we propose an automatic triple-attention dermoscopic image segmentation framework based on deep CNN, AG, spatial, and channel attention modules. The information extracted from the encoded paths is used for gating using AG attention in skip connections, disambiguating irrelevant and noisy responses. The spatial attention module improves the performance of deep networks by capturing the spatial correlation between features. Channel attention improves the representation of the network by capturing explicit relationships between convolutional channels via contextual gating mechanisms [18]. We evaluated the proposed network on three datasets: ISIC-2016 [19], ISIC-2017 [20] and PH2 [21]. The experimental results showed that the new network exhibited good performance.

The main contributions of this paper are as follows:

(1) A new Attention Gate, Spatial and Channel Attention U-Net (ASCU-Net) model was proposed for the accurate segmentation of skin lesions in dermoscopic images. A convolutional multi-attentive module was used to extract the image features and generate resultant maps of skin lesion segmentation.
(2) The multiple attention learning mechanism of the triple attention decoding block was ingeniously designed. The module embeds AG, spatial, and channel attention modules to further improve the feature representation capability, and the U-Net network built on this module significantly improved the performance of skin lesion segmentation. The effectiveness of the triple attention decoder block was verified by an ablation study.
(3) The performance of the ASCU-Net segmentation method was compared with other algorithms on the ISIC-2016 [19] and ISIC-2017 datasets [20], with significant improvements in six evaluation metrics including accuracy, sensitivity, specificity, precision, dice coefficient, and Jaccard index. In addition, to verify the reliability and applicability of the network, the network trained on the ISIC-2017 dataset was put to test on another publicly available dataset named PH2 [21].

The remainder of this paper is organized as follows. The next subsection reviews the related work. Section 2 describes our architecture. Section 3 describes the three datasets used, along with our results. Section 4 provides a discussion of the proposed network, and a final section presents our conclusions.

### 1.2. Related Works

In this section, we briefly review the CNN-based skin lesion segmentation architectures and the existing methods relevant to this work.

#### 1.2.1. Skin Lesion Segmentation

Skin lesion segmentation is a technique for detecting the location and boundaries of clinical image lesions. Traditional algorithms for skin lesion segmentation mainly include threshold-based [22], gradient vector flow methods [23], region growth [24], segmentation methods, and morphology [25], and model [26] based segmentation methods. In recent years, deep learning has shown excellent performance in the field of image processing, and CNN [9]-based segmentation methods have been applied for the first time in the field of image segmentation, with impressive results in skin lesion segmentation [27,28].

Yuan et al. [29] designed a new loss function to optimize the Jaccard distance-based skin lesion segmentation task. Yu et al. [30] proposed a full convolutional residual network (FcRN) for end-to-end training and achieved better segmentation results. Song et al. [31] proposed a dense residual attention network focusing on the fixation receptive field and alleviation of gradient vanish, and Sulaiman et al. [32] used dilation and dense block convolution techniques to integrate multi-scale and global contextual information for improved U-Net networks for skin lesion segmentation. Lei et al. [33] used generative adversarial networks (GANs) to enhance the segmentation of skin lesions. Bi et al. [34] proposed a multi-stage fully convolutional network (FCN), which combined low-level appearance information with high-level semantic information hierarchies. The dsNet proposed by Hasan et al. [35] learns distinguishing features in pixel space projected onto different stages of the encoder and uses separable convolution in a depth-wise separable convolution instead of standard convolution. The skin lesion network proposed by Adegun et al. [36] integrates an encoder–decoder full convolutional network, dense block, and conditional random field (CRF) modules, which are connected by cascading strategies and transition layer merging to reduce model complexity while improving performance. Although existing deep learning methods have shown some performance in skin lesion segmentation, boundary segmentation of high-precision medical images still faces challenges.

#### 1.2.2. Overview of U-Net Architecture

**Figure 2:**
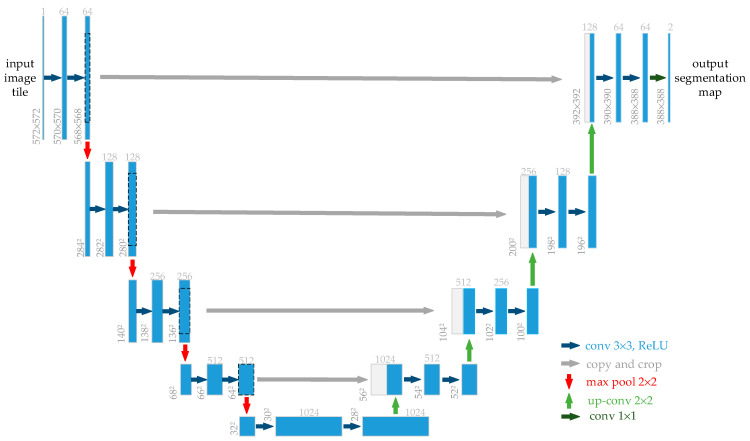
The original U-Net model [9].

Similar to FCN [37] and SegNet [38], Wang et al. proposed the U-Net [9] network for medical image segmentation in 2015 (as shown in Figure 2). U-Net is a neural network with symmetric encoders and decoders, a structure that has shown excellent performance in the field of medical imaging. The U-Net consists of contracted paths for capturing feature information and symmetric extended paths for enabling localization. The middle of the U-Net uses skips connections from encoders to decoders of similar resolution to pass high-resolution information throughout the network. Perhaps, the most ingenious aspect of the design of the U-Net architecture is the skipping of connections. These spatial features, which are lost due to pooling operations, can be retrieved by the network skipping connection layer [39]. In addition, a number of improved models based on the U-Net structure have been proposed to further enhance the reliability of computer-aided medical image diagnostic tasks.

Based on U-Net, Wei et al. [40] proposed an Att-DenseUnet network combining densenet and attention mechanisms with U-Net and achieved good results in skin lesion segmentation. Ibtehaz et al. [41] analyzed the U-Net model in depth and proposed a novel U-Net architecture, MultiResUNet, which has been used to good effect in biomedical image segmentation. Inspired by the state-of-the-art improved U-Net networks that have been proposed, we took into account the ability of the up-sampling process to extract deep features of the image and the ability of hopping joints to deliver high-resolution information, recovering spatial information that is lost due to pooling operations. Our proposed ASCU-Net network builds on the original U-Net network by incorporating different types of attentional mechanisms in the skipping connection layer and in the up-sampling module, respectively, to perform well in the skin lesion segmentation task.

#### 1.2.3. Attention Mechanism

Attention mechanisms play a crucial role in human perception [42,43,44]. Attention mechanisms allow humans to selectively focus on key information while ignoring other irrelevant information. Through the attention module, deep CNN can accelerate the learning process, extract more critical and discriminative features for the target task, enhance the robustness of the network model, and be more adaptable to small training datasets.

**Figure 3:**
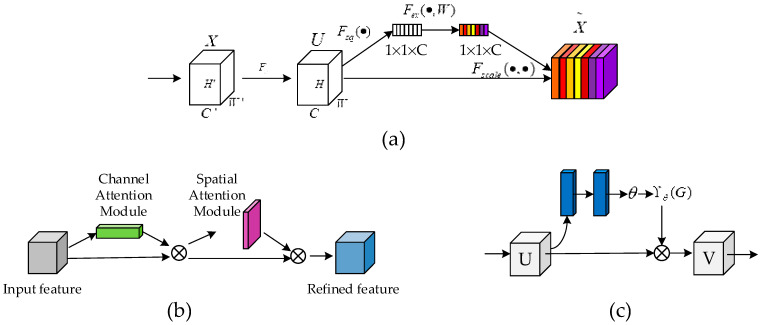
Structure of several typical attentional networks (from left to right SENet [18], CBAM [47] and SpatialNet [48], respectively). CBAM (convolutional block attention module), where (**a**) the main focus is on channel relationships between image input features, (**b**) the main focus is on integrating spatial and channel attention, and (**c**) the main focus is on spatial relationships between image features.

The attention mechanism was first proposed by the Google Deep Mind team while performing an image classification task, thus kicking off a wave of research on the attention mechanism [45]. Kaul et al. [46] proposed a method for incorporating attention into a FCN, FocusNet, which performs medical image segmentation from a feature map generated by a separate convolutional autoencoder. Hu et al. [18] proposed that SENet adaptively recalibrates channeled feature responses by explicitly modeling the interdependencies between channels. Later, Woo et al. [47] further extended the squeeze-and-excitation module in SE-Net. The convolutional block attention module (CBAM) module proposed by the authors is a lightweight general-purpose module. It uses almost no computational resources and is able to perform adaptive feature refinement based on a given intermediate feature map. Figure 3 illustrates the structure of several typical attention modules in a network structure. However, all of the above approaches start from a single focus. As skin lesions can have blurred boundaries, uneven color distribution, and irregular shapes, it is difficult to perform well in skin lesion segmentation tasks by relying on a single attentional mechanism or a two-dimensional integrated attentional mechanism alone.

In response to the above issues, we proposed the triple attention model ASCU-Net, which was developed as an extension to U-Net and showed excellent performance. Our work combined the recent advances in the trainable attention gate that extracts important features from contextual information by Oktay et al. [10], the adaptive reanalysis of channel feature responses through the interdependence of squeeze and excitation modules proposed by Hu et al. [18,49], the spatial attention for highlighting regions of interest, and suppressing background clutter proposed by Jetley et al. [50]. The effectiveness of the network structure in the segmentation of skin lesions was verified by extensive ablation and comparison experiments.

## 2. Materials and Methods

**Figure 4:**
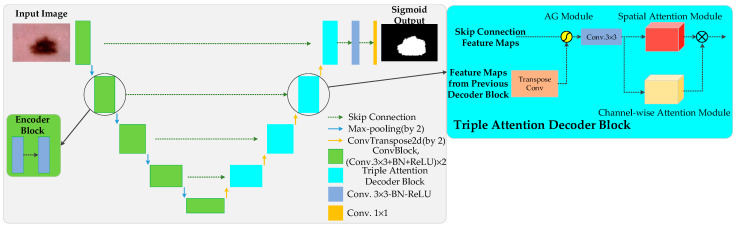
Diagram of the proposed ASCU-Net. AG, attention gate.

Inspired by U-Net [9], attention U-Net [10], spatial attention module (SAM) [48], Squeeze-and-Excitation Networks (SENet) [18] and Shape Attentive U-Net (SAUNet) [16], we proposed ASCU-Net (Figure 4). We describe all parts of the network in detail in the following subsections.

### 2.1. Proposed ASCU-Net Architecture

Figure 4 shows the proposed ASCU-Net with a network structure similar to that of a U-shaped encoder–decoder. Each step of the encoder and decoder consists of a structured convolution block and a triple attention decoder block, respectively. Each convolutional block consists of a convolutional layer (ConvBlock), batch normalization (BN) layer, and rectified linear unit (ReLU). In the encoder path, the network doubles the number of feature channels with each down-sampling step. This corresponds to an up-sampling of the 2 × 2 transpose convolution and a halving of the number of feature channels in the decoder path. The encoder and decoder are connected by skips between the feature maps of the corresponding layers before a structured triple attention decoder block is performed. Skin lesion output segmentation maps are generated at the last layer, after 1 × 1 convolution and application of sigmoid activation functions.

### 2.2. Triple Attention Decoder Block

**Figure 5:**
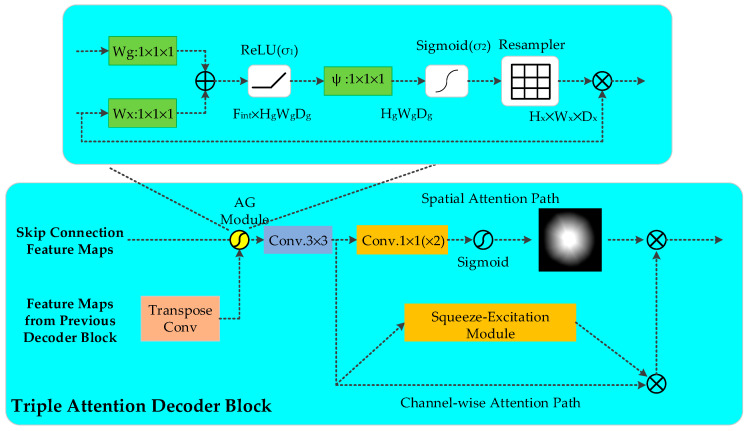
Triple attention decoder block. The proposed attentional decoding module consists of three modules: the AG module for skipping the connection layer to suppress irrelevant information, the spatial attention module, and the channel attention module for fusing the input features together to improve the performance of the network.

The up-sampling (decoding) process fuses the feature map information output from the encoder module via skipping connections as well as capturing additional contextual and spatial information of the feature map from the low-resolution decoder block. We propose the triple attention decoder block (Figure 5), which is a dual attention decoder block consisting of spatial attention and channel attention after AG processing on the concatenated feature map and a standard normalized 3 × 3 convolution operation. The three new components select regions based on contextual information and weights, capture spatial correlations between features, and focus channel attention on channel relationships to improve performance, as demonstrated by Hu et al. [18]

#### 2.2.1. Attention Gate

AG in the U-Net model was first proposed by Oktay et al. [10]. The AG attention module adaptively adjusts and automatically learns to focus on the different shapes and sizes of the target structures in medical images. The model strained with AG implicitly learns to highlight salient features useful for a specific task while suppressing irrelevant regions in an input image.

A diagram of the proposed additive AG is shown in Figure 5. AG selects spatial regions by analyzing the contextual information and activation provided by the gating signal (*g*) collected from coarser scales. The input features ($x^{l}$) are scaled according to the attention coefficients (*α*) of the resampled grids, which are completed using trilinear interpolation. The attention factor $\alpha_{i}\in [0,1]$ is used to identify significant image areas and to determine the focal area. The output of AG is the multiplication of the elements of the input feature mapping and the attention factor: ${\hat{x}}_{{}_{i,c}}^{l}=x_{{}_{i,c}}^{l}\cdot \alpha_{{}_{i}}^{l}$. In the default setting, a single scalar focus value is calculated for each pixel vector $x_{{}_{i}}^{l}\in R^{F_{l}}$, where $F_{l}$ corresponds to the number of feature maps in layer l. A gating vector $g_{i}\in R^{F_{g}}$ is used for each pixel to determine focus regions. Additive attention is formulated as follows:

(1) $$q_{att}^{l}=\psi^{T}(\sigma_{1}(W_{{}_{x}}^{T}x_{{}_{i}}^{l}+W_{{}_{g}}^{T}g_{i}+b_{g}))+b_{\psi }$$

(2) $$\alpha_{{}_{i}}^{l}=\sigma_{2}(q_{att}^{l}(x_{{}_{i}}^{l},g_{i};\Theta_{att}))$$

where $\sigma_{2}(x_{i,c})=\frac{1}{1+exp(-x_{i,c})}$ corresponds to the sigmoid activation function and $\sigma_{1}$ to the ReLU function. The linear transformation $W_{x}\in R^{F_{l}\times F_{\mathrm{int}}},W_{g}\in R^{F_{g}\times F_{\mathrm{int}}},\;\psi \in R^{F_{\mathrm{int}}\times 1},$ and the bias term $\psi \in R^{F_{\mathrm{int}}\times 1},\;b_{\psi }\in R$ form a set of $\Theta_{att}$ parameters, which characterize the AG. The linear transformation is calculated using a 1 × 1 × 1 convolution in the channel direction of the input tensor. The concatenated features $x^{l}$ and g linearly mapped to a $R^{F_{\mathrm{int}}}$ dimensional intermediate space is called vector-based connected attention.

In order to eliminate noisy and irrelevant responses from skipped connections, gating is determined by the relevant information extracted from the coarse scale. In addition, AG only performs operations to merge relevant activations before the connection operation, filtering neuronal activations for forward transmission as well as for backward transmission. After the extraction and fusion of complementary information from each sub-AG coding and decoding path, the output of the skipped connections is obtained. Similar to non-local blocks [51], AG are linearly transformed without any spatial support, and down-sampling to the gated signal reduces the resolution of the input feature map, thus reducing the parameter and computational resource consumption of the network model.

#### 2.2.2. Spatial Attention Module

The spatial attention module (SAM) has been introduced into convolution neural networks as part of the attention module and has shown good performance in classification and detection tasks [47]. Spatial attention is focused on positional information between images, which depicts the spatial relationship between the input features. Formally, the input feature $F\in R^{H\times W\times C}$, where *H*, *W*, and *C* denote the height, width, and number of channels of the image of the entry spatial attention path, respectively. The normalized 1 × 1 convolution and the 1 × 1 convolution make up the spatial attention module. The number of channels is reduced to half after the first convolution, and subsequent convolution reduces the number of channels to 1. $F_{s}^{\prime }$ is obtained by mapping the pixel values in a single channel to the range of [0, 1] via the sigmoid function. In order to perform an element-wise multiplication of the dimension $F_{c}$ from the channel-wise attention path output and the dimension $F_{s}$ from the spatial attention path output, $F_{s}^{\prime }$ is then stacked channel-wise C times to obtain $F_{s}$. In short, the output feature of the spatial attention module is calculated as:

(3) $$F^{s}=\sigma (f^{1\times 1}(\mathrm{ReLU}(BN(f^{1\times 1}(F)))))$$

where $f^{1\times 1}(\cdot )$ represents a convolution operation with a filter size of 1 × 1, BN(⋅) represents batch normalization, and σ(⋅) represents the sigmoid function.

#### 2.2.3. Channel Attention Module

The squeeze and excitation modules form the channel attention module (CAM), which generates a scaling factor of [0, 1] for each channel (i.e., channel attention) of the skipping connection. The $F_{c}$ is the skipping connection profile from each channel scaled according to the scaling factor generated by the respective CAM.

The CAM first performs a squeeze operation. The module generates channel descriptors by using global average pooling (GAP) and aggregates the feature map input to the CAM in the entire channel context. We used $\gamma_{d}^{up}=[y_{1}^{up},y_{2}^{up},\ldots ,y_{F}^{up}]$, where $y_{f}^{up}\in R^{W\times H}$, as the input data to the channel attention module. The global average pooling is performed as follows:

(4) $$s_{f}=F_{sq}(y_{f}^{up})=\frac{1}{H\times W}\sum_{m}^{H}\sum_{n}^{W}y_{f}^{up}(m,n)$$

where $F_{sq}$ is the spatial squeeze (GAP) function; $y_{f}^{up}$ is the spatial position of the $f^{th}$ channel; and *H* × *W* correspond to the height and width dimensions, respectively, of this channel. Briefly, $s_{f}$ is generated from each two-dimensional feature map by GAP compression. The second step of the channel attention module is motivation. It captures the dependencies between channels based on the global information embedded in the first step. Non-exclusive relationships and non-linear interactions between channels can be learned by this function [18]. The SE block shown in Figure 5 contains two full-connection (FC) layers, where the input vectors are sequentially encoded in the shapes of $1\times 1\times \frac{F}{r}$ and 1×1×F. The output of the SE block is shown in Figure 5. The output of the final SE block is represented as:

(5) $$C_{s}=F_{se}(z;S)=\sigma (S_{2}\delta (S_{1}z))$$

where $S_{1}$ and $S_{2}$ are the parameters for the first FC layer and the second FC layer, respectively. *δ* is a rectified linear unit (ReLU), and *σ* refers to the sigmoid function. In addition, r is the reduction ratio.

#### 2.2.4. Channel and Spatial Attention

In the triple attention decoder block, we refer to the channel and spatial attention as two independent modules. The feature map transmitted from the AG is processed by the channel and spatial attention modules, respectively, after a 3 × 3 convolution operation, and the channel and spatial attention are fused as the output of the triple attention decoder block, as shown in Figure 5. Considering that spatial attention captures spatial relationships between features and improves the segmentation performance of the network, channel attention is able to learn non-linear interactions and non-repulsion relationships between channels. Therefore, in the design of the network structure, the input features are first subjected to a convolutional operation to increase the nonlinear representation of the network and to reduce the parameters while reducing the computational cost. Spatial attention and channel attention are used as two parallel routes to capture regions of interest in both the spatial and channel dimensions simultaneously and to fuse the output as input for the next decoding operation. Output F can be obtained as follows:

(6) $$F=F_{c}⊗(F_{s}+1)$$

The operator ⊗ represents the Hadamard product. It includes +1, so the spatial attention that is initially in the range [0, 1] can only amplify features, not zero out features that may be valuable in subsequent convolutions.

### 2.3. Loss Function

The cross-entropy loss function is often used in image segmentation and classification tasks as cross-entropy measures the difference in information between the ground truth and prediction distributions. Typically, the average number of bits of the coding length required to identify a sample by the ground truth distribution p is used as a measure of the cross-entropy definition between the ground truth distribution p and the probability distribution q.

The cross-entropy loss in image segmentation tasks is usually calculated as the average cross-entropy of all pixels. Let Ω denote the domain of all pixels of height h, width w, and class K. $x\in M_{h\times w\times K}$({0, 1}) and $\hat{x}\in M_{h\times w\times K}$([0, 1]) are expressed as the ground truth mono-heat matrix encoding the ground truth class of each pixel and the predicted probability matrix of each individual pixel, respectively. The cross-entropy loss can be deduced from the following formulae:

(7) $$H_{CE}(\hat{x},x)=\frac{1}{\left|\Omega \right|}\sum_{j}^{\Omega }-((1-x_{j})\log(1-{\hat{x}}_{j})+x_{j}\log({\hat{x}}_{j}))$$

## 3. Experiments and Results

We performed the evaluation of the proposed method using three datasets: ISIC-2016, ISIC-2017, and PH2. For the PH2 dataset, all results were obtained from deep learning model testing trained on the ISIC-2017 segmentation training set. ISIC-2016 and ISIC-2017 were trained and tested using their respective training and test sets, respectively. In addition, all three datasets provided original images and paired skin lesion segmentation maps annotated by specialist dermatologists.

### 3.1. Performance Evaluation Metrics

In this paper, we evaluated the segmentation performance of different networks using common criteria for skin lesion segmentation in the ISBI 2016 and 2017 Lesion Segmentation Challenge [23], and PH2 dataset [21] including accuracy (AC), sensitivity (SE), specificity (SP), precision (PC), dice coefficient (F1), and Jaccard index (JS). Accuracy is an assessment of the overall segmentation performance of the lesion image [52]. The number of correctly segmented skin lesion pixels is reflected by the sensitivity [52,53]. Specificity is defined as the proportion of non-lesion areas that are correctly segmented. Precision indicates the number of true correct ones as a percentage of the overall result. The overlap between the predicted results and the ground truth is defined as the dice coefficient, which is an index of similarity for image segmentation, and the Jaccard index is an evaluation measure of the intersection ratio between the resulting segmentation results and the ground truth mask [54]. The metrics for evaluating segmentation results are defined as:

(8) $$\begin{array}{l} AC=\frac{TP+TN}{TP+TN+FP+FN}\\ SE=\frac{TP}{TP+FN}\\ SP=\frac{TN}{TN+FP}\\ PC=\frac{TP}{TP+FP}\\ F1=2*\frac{PC*SE}{PC+SE}\\ JS=\frac{\left|GT\cap SR\right|}{\left|GT\cup SR\right|} \end{array}$$

where *TP*, *TN*, *FP*, and *FN* are the numbers of true positive, true negative, false positive, and false negative, respectively. *TP* is the number of pixels that are in fact positive samples (areas of interest) and have been judged to be positive samples. *TN* is the number of pixels that are in fact negative samples (skin areas) and have been judged to be negative samples. *FP* is the number of negative sample pixels that have been misclassified as positive samples. *FN* is the number of positive sample pixels that have been misclassified as negative samples.

### 3.2. Experimental Setups

#### 3.2.1. Dataset

We used three dermoscopic image datasets to assess the proposed network and compare it with other methods, the ISIC-2016 challenge dataset [19], ISIC-2017 challenge dataset [20] and PH2 dataset [21]. The International Skin Imaging Collaborative (ISIC) provides expertly annotated digital skin lesion image datasets from around the world to facilitate computer-aided diagnosis (CAD) of melanoma and other skin diseases and to facilitate automated and efficient computer diagnosis [20]. The ISIC-2016 challenge dataset contains 900 training images and 379 test images. The ISIC-2017 dataset is a dataset published by ISIC and used for the Skin Lesion Segmentation Challenge. The challenge dataset contains 8-bit RGB dermoscopic images with image sizes ranging from 540 × 722 to 4499 × 6748 pixels. It provides 2000 training images and individual sets of 150 and 600 images, respectively, for validation and testing. The PH2 dataset [21] is a database of dermoscopic images proposed for segmentation and classification, which were organized by a joint collection from the dermatology service of Hospital Pedro Hispano in Matosinhos, Portugal, and the University of Porto. This dataset contains a total of 200 8-bit RGB color skin images with a resolution of 768 × 560 pixels including 80 common nevi, 80 atypical nevi, and 40 melanomas. We used this dataset as an additional test set for the deep learning model trained on the ISIC-2017 split training set. Table 1 summarizes the sources and other specific information about the three datasets. In addition, all three datasets provided raw images and paired skin lesion segmentation maps annotated by a specialist dermatologist. To enhance the generalizability and robustness of the model, the training dataset was augmented with data augmentation using horizontally and vertically flipped randomly generated samples.

#### 3.2.2. Implementation Details

We implemented our network using Pytorch on a GPU server with Intel I9-10900X CPU @3.70 GHz, 32 GB DDR4 RAM, and Nvidia GeForce TITAN RTX. All training and tests were performed in the same hardware environment. The operating system used for the experiments was Ubuntu 16.04, using Python 3.5 as the programming language and the Pytorch 1.5.0 framework for the design of the neural network structure and the debugging of the model. The network uses the AdamW optimizer for end-to-end training. We trained 200 epochs with the initial learning rate set to 0.0002, momentum parameters b1 = 0.9, b2 = 0.999, and batch size set to 8. The layer-by-layer transfer of network training losses and updating of parameters relies on back-propagation algorithms.

### 3.3. Comparative Experiment

#### 3.3.1. Comparison on the ISIC-2016 Dataset

**Figure 6:**
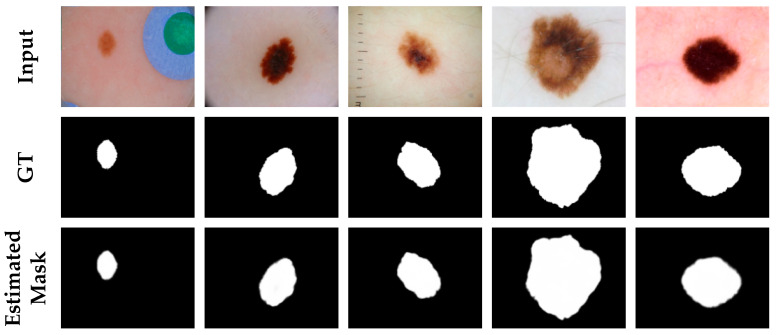
Segmentation results of ASCU-Net on the ISIC-2016 dataset. The first row is the original image. The second row is the skin lesion segmentation ground truth (GT). The third row is the visual segmentation result of the test set of the proposed network.

We trained and evaluated the proposed network on the ISIC-2016 dataset. Table 2 summarizes the quantitative results comparing our proposed method with other methods on the ISIC-2016 dataset. As can be seen from the table, our proposed network achieved satisfactory results. In particular, the assessment metrics of accuracy, sensitivity, dice coefficient, and Jaccard index differed by an order of magnitude, which was sufficient to show that the improvement in the performance of our network compared to other networks was significant. This is despite the fact that the specification metrics that we used were slightly inferior to those used in other methods, which means that for images with large lesion areas, the model does not perform as well as for images with normal lesion areas. However, the combined performance of the six metrics was still a strong indication that our proposed network was sufficiently successful. Figure 6 shows a visualization of the skin lesion segmentation of our proposed network. The effectiveness of the algorithm can also be visualized in experimental renderings.

#### 3.3.2. Comparison on the ISIC-2017 Dataset

**Figure 7:**
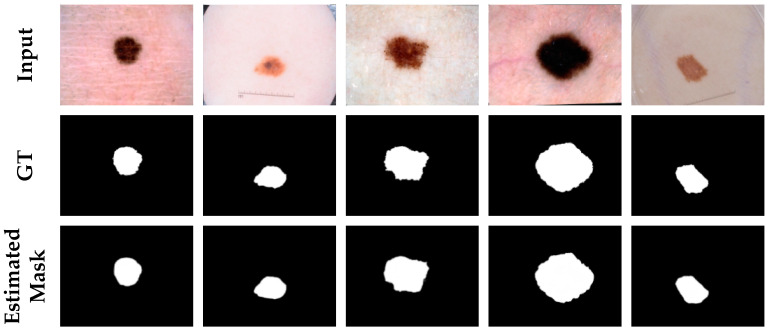
Segmentation results of ASCU-Net on the ISIC-2017 dataset. The first row is the original image. The second row is the skin lesion segmentation ground truth (GT). The third row is the visual segmentation result of the test set of the proposed network.

In this section, we further trained and tested the proposed network on the ISIC-2017 dataset. In Table 3, a quantitative comparison between the segmentation performance of the proposed network and other methods is presented. Due to the presence of more images that are difficult to segment accurately in this dataset, the metric scores of other networks in this dataset are hardly satisfactory, but our proposed network still achieves satisfactory evaluation metrics. In particular, the precision, dice coefficient, and Jaccard index, which differed by an order of magnitude, indicated that our proposed method was sufficiently successful to achieve satisfactory results in the segmentation of skin lesions. Figure 7 shows the output of the visualization of the proposed network in this dataset of partial images of skin lesion segmentation. The results also showed that the performance of our proposed network was excellent.

#### 3.3.3. Comparison on the PH2 Dataset

**Figure 8:**
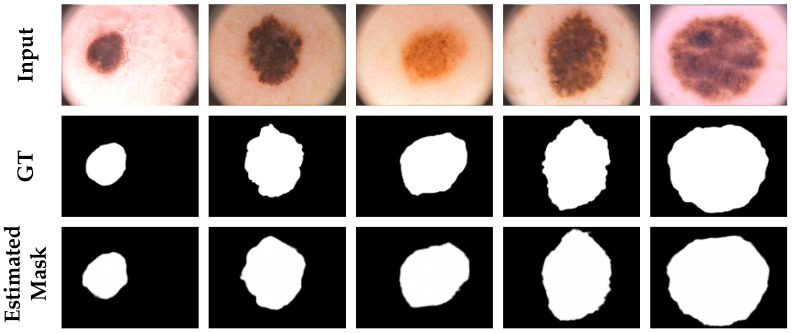
Segmentation results of ASCU-Net on the PH2 dataset. The first row is the original image. The second row is the skin lesion segmentation ground truth (GT). The third row is the visual segmentation result of the test set of the proposed network.

In order to illustrate the generalizability and robustness of our proposed network, we used the model trained on the ISIC-2017 dataset for the evaluation of the metrics in the PH2 dataset. Table 4 presents a comparison between the proposed network and the quantitative results obtained by the other methods in the PH2 dataset. The ASCU-Net had better accuracy, sensitivity, precision, dice coefficient, and Jaccard index compared to those obtained using other methods, which means that our proposed method had a higher overall pixel-level segmentation performance. Excellent segmentation results could be obtained in the segmentation of skin lesions. Figure 8 visually shows the segmentation results for the parts of the proposed network that performed well in this dataset. Thus, by combining the experimental segmentation results with the evaluation metrics, the results showed that our proposed triple-attention idea was successful not only in improving the performance of the network, but also in providing good generalizability. 

### 3.4. Ablation Experiment

To illustrate the effectiveness of our proposed triple attention U-Net network, we set up ablation experiments. In the ablation experiments, all network training and tests were performed in the same hardware environment using the ISIC-2017 dataset.

AG, spatial, and channel attention are the main components to improve segmentation performance. The ablation experiments with different attention decoding modules were designed to illustrate the role played by triple attention decoding blocks in networks. Using an attention-free encoder-decoder network as a benchmark model, different attention mechanisms were added to the attention decoder block and compared with our triple attention decoder block.

In Table 5, we compared the performance of eight modalities of skin lesion segmentation: AG+spatial+channel (ours), No-attention, single-AG, single-spatial, single-channel, AG+channel, AG+spatial, and spatial+channel, respectively. As can be seen from this table, while single attention or a combination of the other two types of attention as an attention decoder block made the network perform better to some extent, our proposed triple attention decoder block only slightly underperformed the other specificity and precision assessment. This means that our proposed network structure had only a slightly poorer specificity and precision compared to other approaches. However, the overall segmentation results of the triple attention decoder block were much higher than those of the other attention decoding modules when considering the combined six evaluation metrics and the final segmentation of the skin lesions. Our proposed network achieved satisfactory results.

The AG attention module is able to analyze contextual information and help the network focus more on local areas by scaling the attention coefficients. This increases the sensitivity of the model to foreground pixels without the need for a complex heuristic algorithm. Spatial attention modules reflect the spatial relationships between features, focus on regions of spatial interest, and make full use of global contextual information. The channel attention module is a SE block that contains two operations: squeeze and excitation. Global features at the channel level are first acquired via a squeeze operation on the global average pool. The excitation operation then captures the inter-channel dependencies of the global information embedded in the first step. Finally, the number of weights is kept the same for the output and the input features.

The combination of AG, spatial, and channel attention modules is a good solution to the low grayscale variation and relatively blurred boundaries of skin lesion images. Our proposed triple attention decoder block improves its ability to recognize representations by stepwise pooling using AG, spatial, and channel attentional learning mechanisms. The attention learning mechanism generates low-level attentional maps using high-level learned features, which greatly improves the segmentation performance of skin lesions, while reducing the complexity of the network model and the consumption of computational resources.

**Figure 9:**
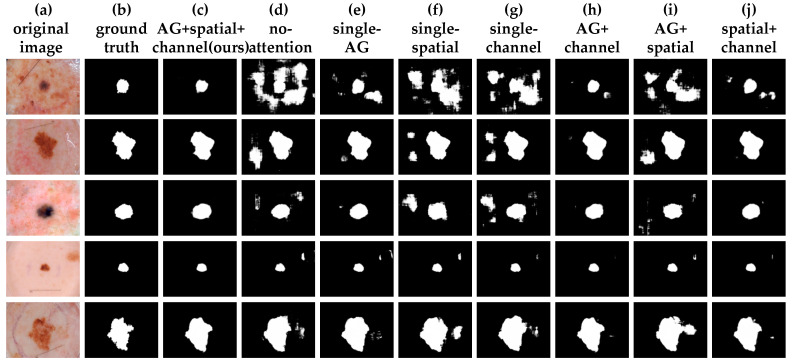
Visualization results of an ablation experiment. From left to right are the original image of the dermoscope, skin lesion segmentation ground truth, AG+spatial+channel(ours), no-attention, single-AG, single-spatial, single-channel, AG+channel, AG+spatial and spatial+channel.

Figure 9 shows a graphical visualization of the results of the ablation experiment, showing five examples of dermoscopic images and the segmentation masks corresponding to the different attention decoding modules. It is clear from the segmentation results shown in Figure 9 that our proposed method had a clearer segmentation of the boundary information than either a single or a combination of any two attentional approaches, resulting in a more focused network on the skin lesion region. It is clear that the triple-attention decoder block had better feature representation than other decoding modules.

## 4. Discussion

Our proposed networks made several modifications to the initial U-Net. Through ablation experiments, we evaluated each modified part of each network and analyzed its impact on the results.

In this work, we started by analyzing of the U-Net architecture, focusing on the impact of the attention mechanism on the network structure, with the hope of finding potential rooms for improvement and enhancing the network performance for skin lesion segmentation of medical images. To enhance the ability of the U-Net network to capture key information about images, we proposed the concept of a triple attention mechanism. We took inspiration from the AG, SENet, and SAM blocks and formulated a compact analogous structure that was lightweight. Combining the fusion of these attention mechanisms, we developed a novel architecture, ASCU-Net. In the decoder path, the triple attention module was selected to implement the decoding process of the network. As can be seen from Table 2, Table 3, Table 4 and Table 5, ASCU-Net with triple attention had better performance. The core idea of the attention mechanism is to focus on the area of interest according to the weights of the attention factors, filtering out the unimportant from the large amount of information, and fusing the small amount of important information together. Therefore, adding gating (AG layer) to the skip connection layer can help the network acquire more important information, which can help the network to increase the performance of deeper models. The module of dual attention during up-sampling is able to acquire feature maps with rich local and semantic information. Spatial attention captures the spatial correlation between features, and channel attention captures the explicit relationship between channels in the convolutional layer through a contextual gating mechanism, assigning a weight (i.e., channel attention) to each channel in the feature map to encode the feature map. The visualized skin lesion segmentation results included in Figure 6, Figure 7, Figure 8 and Figure 9 show that ASCU-Net enables a finer output of skin lesion segmentation, not only by focusing on information about edge features in the image, but also by capturing key information about the input image features using multiple attention mechanisms. 

Compared to the original U-Net, not only did the segmentations generated by ASCU-Net attain higher scores in the evaluation metrics, but they were also visually more similar to the ground truth. Furthermore, on very challenging images, U-Net tended to over-segment, make false predictions, and even miss the objects completely. In contrast, in the experiments, ASCU-Net showed much higher reliability and robustness. ASCU-Net was able to detect finer details and was highly adaptable to image segmentation with a lot of perturbations. Although our proposed method did not deliver the best segmentation performance compared to the state-of-the-art methods, the algorithm still achieved acceptable segmentation results without pre- and post-processing. ASCU-Net fuses multiscale information captured by the three attentional mechanisms to effectively improve the segmentation performance of the network. The aim of this paper was the effectiveness of the three attention mechanisms for skin lesion segmentation, and our experimental results provide strong evidence for the hypothesis presented in the paper. 

Therefore, we believe that our proposed ASCU-Net architecture can be the potentially successful architecture. There are several branches of future research directions. First, we will further integrate information on multi-scale attentional features by fine-tuning the hyper-parameters of the network in the hope of further improving the performance of the network through experiments. Second, additional pre-processing techniques can be incorporated such as removing hair follicles and color normalization can improve the performance of these algorithms. Finally, conducting research related to simple post-processing methods (e.g., selecting the largest segmented object in a segmentation mask) would also help to improve the performance of the network. Furthermore, while this study focused only on the overall approach to the segmentation task for skin lesion datasets, our work is equally applicable to other medical imaging applications such as lung segmentation, CT image segmentation, or retina blood vessel segmentation. We believe that applying our model to other medical imaging applications and combining it with appropriate pre- and post-processing stages will enrich the applicability and feasibility of the network in the medical domain and allow us to develop better segmentation methods for different medical image applications.

## 5. Conclusions

We proposed a triple-attention-based image segmentation algorithm for skin lesions. The results showed that our network was able to capture more distinguishing information by adding AG modules in the skip connections and channel attention and spatial attention modules in the decoding paths. The experimental results on three public benchmark datasets showed that the network had higher gains in semantic segmentation and achieved more accurate segmentation results than the original U-Net network and other improved U-Net networks.

## Figures and Tables

**Table 1:** The specific information of the ISIC-2016 dataset, ISIC-2017 dataset, and PH2.

Datasets	ISIC-2016	ISIC-2017	PH2
Obtained from	ISIC	ISIC	Hospital Pedro Hispano, Portugal.
Total number	1279	2750	200
Train/Test number	900/379	2150/600	0/200
Resolution (pixel)	576 × 768 to 2848 × 4288	540 × 722 to 4499 × 6748	560 × 768
Augmentation methods	Horizontal-vertical flip		Only for testing

**Table 2:** Performance comparison between the proposed network and other methods on the ISIC-2016 dataset.

Methods	Performance Evaluation Metrics					
	AC	SE	SP	PC	F1	JS
U-Net [9]	0.943	0.907	0.962	0.895	0.887	0.812
Attention U-Net [10]	0.944	0.908	0.963	0.890	0.886	0.811
U-Net++ [55]	0.943	0.903	0.964	0.901	0.889	0.815
Recurrent U-Net [56]	0.937	0.896	0.965	0.884	0.874	0.793
Ours	0.954	0.927	0.961	0.915	0.908	0.845

**Table 3:** Performance comparison between the proposed network and other methods on the ISIC-2017 dataset.

Methods	Performance Evaluation Metrics					
	AC	SE	SP	PC	F1	JS
U-Net [9]	0.913	0.762	0.976	0.887	0.781	0.687
Attention U-Net [10]	0.913	0.765	0.976	0.889	0.783	0.692
U-Net++ [55]	0.912	0.749	0.979	0.900	0.777	0.685
Recurrent U-Net [56]	0.905	0.816	0.953	0.782	0.754	0.643
Ours	0.926	0.825	0.965	0.897	0.830	0.742

**Table 4:** Performance comparison of the proposed network and other methods on the PH2 dataset.

Methods	Performance Evaluation Metrics					
	AC	SE	SP	PC	F1	JS
U-Net [9]	0.910	0.885	0.959	0.899	0.873	0.794
Attention U-Net [10]	0.916	0.899	0.958	0.895	0.880	0.802
U-Net++ [55]	0.909	0.883	0.960	0.900	0.873	0.794
Recurrent U-Net [56]	0.919	0.926	0.945	0.867	0.882	0.800
Ours	0.943	0.960	0.937	0.877	0.909	0.842

**Table 5:** Performance comparison of different attentional mechanisms on the ISIC-2017 dataset.

Methods	Attention Mechanism			Performance Evaluation Metrics					
	AG	Spatial Attention	Channel Attention	AC	SE	SP	PC	F1	JS
No-attention	×	×	×	0.912	0.764	0.973	0.881	0.778	0.681
single-AG	√	×	×	0.923	0.795	0.976	0.910	0.816	0.726
single-spatial	×	√	×	0.911	0.757	0.976	0.889	0.775	0.681
single-channel	×	×	√	0.910	0.755	0.977	0.887	0.774	0.678
AG + channel	√	×	√	0.925	0.819	0.973	0.897	0.825	0.737
AG + spatial	√	√	×	0.910	0.753	0.978	0.892	0.776	0.682
Spatial + channel	×	√	√	0.924	0.798	0.977	0.914	0.822	0.734
Ours	√	√	√	0.926	0.825	0.965	0.897	0.830	0.742

## Data Availability

The data used for training and test-set ISIC-2016, ISIC-2017 are available at: Available online: https://challenge.isic-archive.com/data#2018. The data used for test-set PH2 are available at: Available online: https://www.researchgate.net/publication/300467916_PH2_A_Public_Database_for_the_Analysis_of_Dermoscopic_Images.
